# Treatment of an Intramammary Bacterial Infection with 25-Hydroxyvitamin D_3_

**DOI:** 10.1371/journal.pone.0025479

**Published:** 2011-10-03

**Authors:** John D. Lippolis, Timothy A. Reinhardt, Randy A. Sacco, Brian J. Nonnecke, Corwin D. Nelson

**Affiliations:** 1 Ruminant Diseases and Immunology Research Unit, National Animal Disease Center, Agricultural Research Service (ARS), United States Department of Agriculture (USDA), Ames, Iowa, United States of America; 2 Department of Biochemistry, University of Wisconsin-Madison, Madison, Wisconsin, United States of America; Ludwig-Maximilian-University, Germany

## Abstract

Deficiency of serum levels of 25-hydroxyvitamin D_3_ has been correlated with increased risk of infectious diseases such as tuberculosis and influenza. A plausible reason for this association is that expression of genes encoding important antimicrobial proteins depends on concentrations of 1,25-dihydroxyvitamin D_3_ produced by activated immune cells at sites of infection, and that synthesis of 1,25-dihydroxyvitamin D_3_ is dependent on the availability of 25-hydroxyvitamin D_3_. Thus, increasing the availability of 25(OH)D_3_ for immune cell synthesis of 1,25-dihydroxyvitamin D_3_ at sites of infection has been hypothesized to aid in clearance of the infection. This report details the treatment of an acute intramammary infection with infusion of 25-hydroxyvitamin D_3_ to the site of infection. Ten lactating cows were infected with ** in one quarter of their mammary glands. Half of the animals were treated intramammary with 25-hydroxyvitamin D_3_. The 25-hydroxyvitamin D_3_ treated animal showed significantly lower bacterial counts in milk and showed reduced symptomatic affects of the mastitis. It is significant that treatment with 25-hydroxyvitamin D_3_ reduced the severity of an acute bacterial infection. This finding suggested a significant non-antibiotic complimentary role for 25-hydroxyvitamin D_3_ in the treatment of infections in compartments naturally low in 25-hydroxyvitamin D_3_ such as the mammary gland and by extension, possibly upper respiratory tract infections.

## Introduction

The relationship between vitamin D status and the ability of that animal's immune system to effectively prevent disease is a topic of much research in both human and veterinary medicine [1]–[5]. Vitamin D, following its conversion to its active form 1, 25-dihydroxyvitamin D_3_ (1,25(OH)_2_D_3_), the active form of vitamin D, is a primary regulator of calcium and skeletal homeostasis [1]. However, additional functions in the immune system became evident in the early 1980s when it was found that 1,25(OH)_2_D_3_ was produced by monocytes in diseased tissue, the vitamin D receptor was identified in immune tissues and some immune functions were shown to be influenced by 1,25(OH)_2_D_3_ [6]–[11]. More that 80 years prior to the demonstration of the role of vitamin D in immune function, cod liver oil or exposure to sun, both sources of vitamin D, were used to treat tuberculosis (Reviewed in: [2], [12]. Then in 1986, Rook and co-workers showed that 1,25(OH)2D3 induced anti-tuberculosis activity in cultured monocytes [13]. Additionally, 1,25(OH)_2_D_3_ has been found to affect monocyte chemotaxis [14] and act as an adjuvant in the production of bacterial-specific antibodies [15]. In 2006, a seminal paper was published by Liu *et. al.* [16] in which they demonstrated that toll-like receptor (TLR) activation of monocytes induced 25-hydroxyvitamin D-1α-hydroxylase (1α -hydroxylase). 1-hydroxylase converts 25-hydroxyvitamin D_3_ (25(OH)D_3_) to the active 1,25(OH)_2_D_3_. 1,25(OH)_2_D_3_ induced the antimicrobial peptide cathelicidin and inhibited the growth of *Mycobacterium tuberculosis*. Furthermore, they showed that cathelicidin induction was compromised when using serum from donors with low 25(OH)D_3._ This suggested that maintaining vitamin D status above that needed for normal calcium homeostasis was required for optimal immune responses via this newly highlighted intracrine pathway.

Associations between serum 25(OH)D_3_ concentrations and optimal immune function is now a subject of significant scrutiny. Levels of serum 25(OH)D_3,_ sufficient for full functionality of the immune system are thought to be higher than levels needed for proper skeletal formation [17], [18]. Using samples collected as part of the National Health and Nutrition Examination Survey, researcher determined the levels of 25(OH)D_3_ in various populations [19]. Their data indicated that in humans only 20–25% of the population has 25(OH)D_3_ levels considered immunologically sufficient (>30 ng/ml) [17], [19]. There is an inverse correlation between serum 25(OH)D_3_ levels and the risk for upper respiratory tract infections [20], tuberculosis [21], and multiple sclerosis [22]. Dietary supplementation of vitamin D has been shown to decrease the risk of relapse in multiple sclerosis patients [23] and decreases the risk of influenza A infections [24]. Together this information indicates an important role of vitamin D in the clearance of infections and containment of inflammation by the body's immune cells.

Mastitis in dairy cattle allows for unique studies of immune cells and the role of vitamin D in modulating the immune system's response to pathogens. First, it is known that intramammary infections activate bovine macrophages found in the milk through the TLR pathways resulting in the upregulation of the expression of the 1α-hydroxylase gene. The expression of 1α-hydroxylase is responsible for the conversion of 25(OH)D_3_ to active hormone 1,25(OH)_2_D_3_ [4]. The production of 1,25(OH)_2_D_3_ leads to changes in gene expression in macrophages isolated from milk of an infected gland [3]. Therefore, the intracrine pathway described in humans [16] is active in the bovine mammary gland macrophages during a bacterial infection, but fails to induce the induction of cathelicidin [4]. A second important aspect of studying the role of vitamin D in mammary gland infections, is that milk is deficient in 25(OH)D_3_. The levels of 25(OH)D_3_ in milk are only 0.3–0.6 ng/ml [25], thus immune cells are devoid of a source of 25(OH)D_3_ after they enter the infected mammary gland. The hypothesis tested in these experiments was that infusion of 25(OH)D_3_ into the mammary gland of a dairy cow infected with *Streptococcus uberis* would reduce the severity of the infection.

## Materials and Methods

### Animals

Ten mid-lactation primiparous Holstein cows at the USDA National Animal Disease Center were used for this study. The National Animal Disease Center animal care and use committee approved all animal-related procedures used in this study (Protocol ARS-4001). Prior to the study, all cows were healthy and bacteria were not detected in their milk prior to the study. Cows were feed a standard ration, which included between 30,000 and 40,000 IU of vitamin D per day. Cows were milked two times a day.

### Infection and Treatment

Mammary gland infection was induced by infusion of approximately 500 cfu of *Streptococcus uberis* strain 0140 (*S. uberis*; a gift from Dr. Max Paape, USDA, Beltsville, MD) suspended in 10 mL of FBS into one quarter of all ten cows. Infected animals were randomly divided into two treatment groups: the first group received 100 ug of 25(OH)D_3_ in 10 ml of FBS in the infected quarter at the completion of each milking, starting at the time of infection and continuing throughout the experiment. The second group received 10 ml of FBS only in the infected quarter at the completion of each milking. Antibiotics were not used during the study.

### Collection of milk, blood and temperature data

Milk samples were aseptically collected from infected quarters at each milking (twice daily) throughout the study. Milk was used for determination of bacterial counts, somatic cell counts, determination of bovine serum albumin (BSA) levels, and 25(OH)D_3_ levels. Milk was serially diluted in sterile phosphate-buffered saline and spread on blood agar plates, then incubated for 24 h at 37°C. Following incubation, plates were examined for bacterial growth and colonies enumerated.

Milk somatic cell counts were determined by sampling milk, adding a preservative, and counting at a Dairy Herd Information Association (DHIA) (Dubuque, IA) approved facility. Bovine serum albumin levels in the milk were measured using a commercial ELISA quantitation kit (Bethyl Laboratories, Montgomery, TX). The kit was used according to manufacturer's protocol and protein concentrations determined using the included standards.

Blood samples (10 ml) were taken by venipuncture of the jugular vein once a day. Serum was obtained by centrifugation. The levels of 25(OH)D_3_ in the serum were determined by a radioimmunoassay [26].

Rectal temperatures were obtained twice a day, at the time of milking.

### Statistical analysis

Data were analyzed as a completely randomized design (JMP version 7 SAS Institute Inc., Cary, NC). Cow served as the experimental unit in the analysis of all data. Effects of treatments on variables (i.e. bacterial counts, rectal temperatures, somatic cell counts, serum albumin, feed intake, milk production) were analyzed with repeated-measures ANOVA. Bacterial and somatic cell counts were log10 transformed prior to analysis. The model included the fixed effects of treatment, time, and treatment x time interaction. Post hoc tests were applied when treatment, time, or treatment x time effects were detected. The values presented for all variables are the means and standard errors of the mean.

## Results

**Figure 1:**
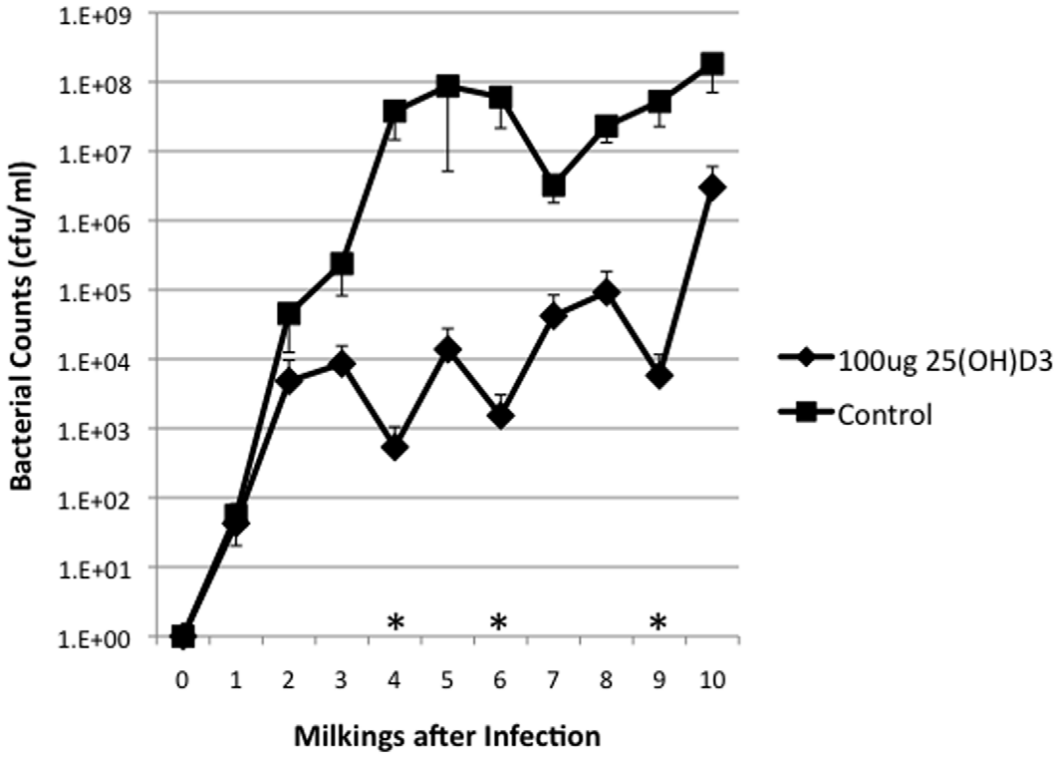
Bacterial Counts in Control and 25(OH)D_3_ Treated Animals. Ten dairy cattle were infused with approximately 500 cfu of *S. uberis* in one quarter of their mammary gland. Five cows were immediately treated with 100 ug of 25(OH)D_3_ in FBS and the remain five cows were treated with FBS alone. Cows were subsequently treated after each milking (twice daily) with 25(OH)D_3_ or FBS for 10 milkings (5 days). Milk sample were isolated from each cow and serially diluted and plated on blood agar plates. Average bacterial counts were determined for 25(OH)D_3_ treated (υ) and control animals(ν). Time points with statistically significant differences are indicated with (*).

All ten animals were successfully infected with *S. uberis*. Establishment of infection was indicated by at least one time point having a bacterial count of greater than 1000 cfu/ml (data not shown). Figure 1 shows the mean bacterial counts of the 25(OH)D_3_ treated group and the control group. There was a significant effect (*P*<0.05) of the 25(OH)D_3_ treatment (figure 1). In addition, there were significant reductions (*P*<0.05) in milk bacterial counts at the fourth, sixth, and ninth milkings in the 25(OH)D_3_ treatment group compared to the control group.

**Figure 2:**
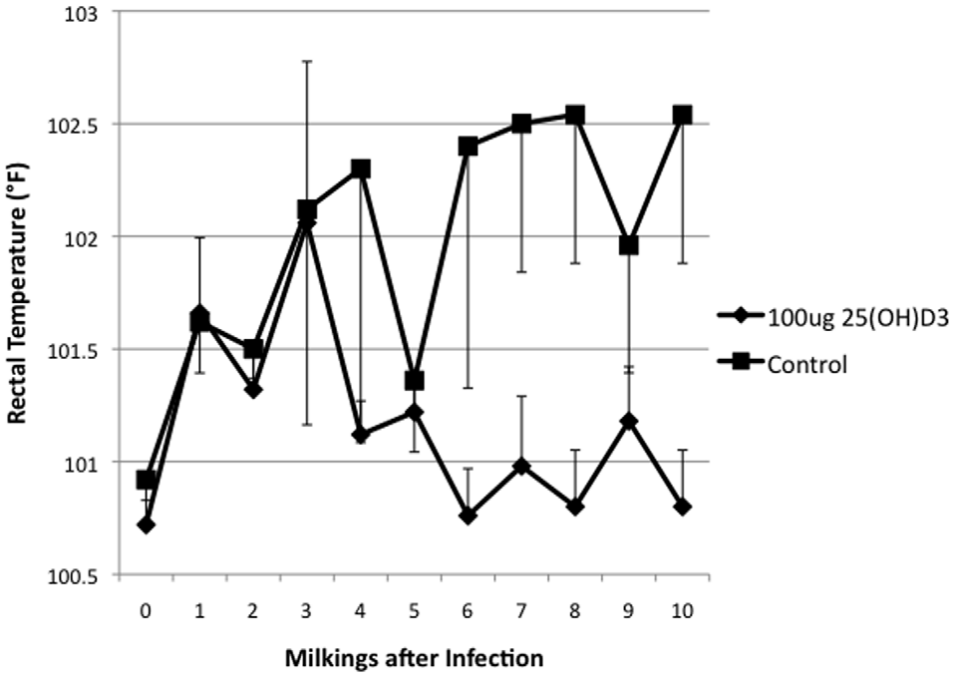
Rectal Temperature in Control and 25(OH)D_3_ Treated Animals. Rectal temperature were taken twice daily, at each milking, and the average was determined for 25(OH)D_3_ treated (υ) and control animals(ν).

**Figure 3:**
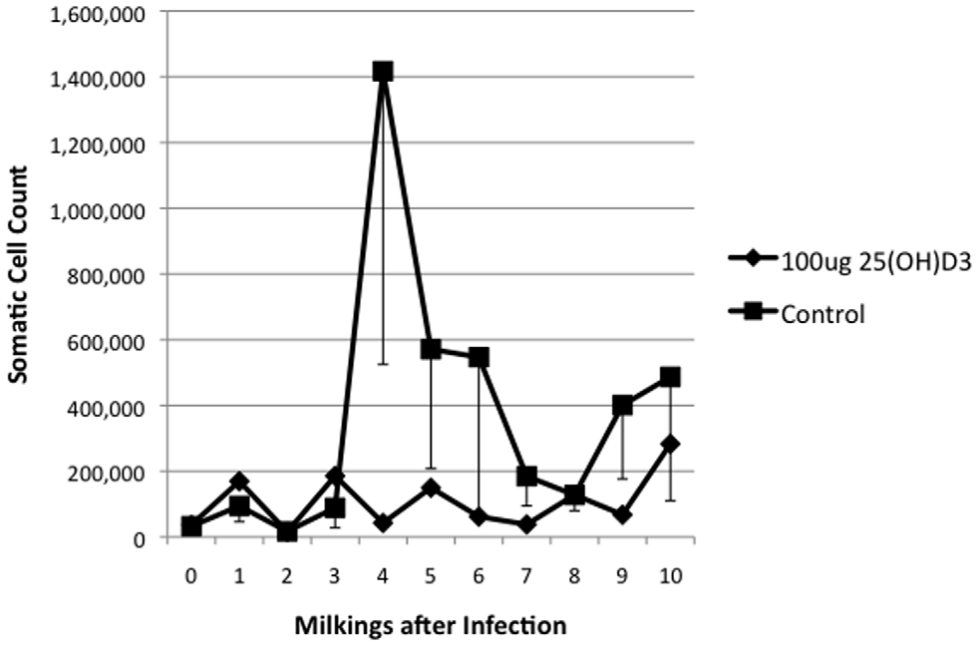
Somatic Cell Counts in Control and 25(OH)D_3_ Treated Animals. Milk samples for somatic cells counts (SCC) were taken at each milking and sent to a DHIA facility for counting. The average SCC were determined for 25(OH)D_3_ treated (υ) and control animals(ν).

**Figure 4:**
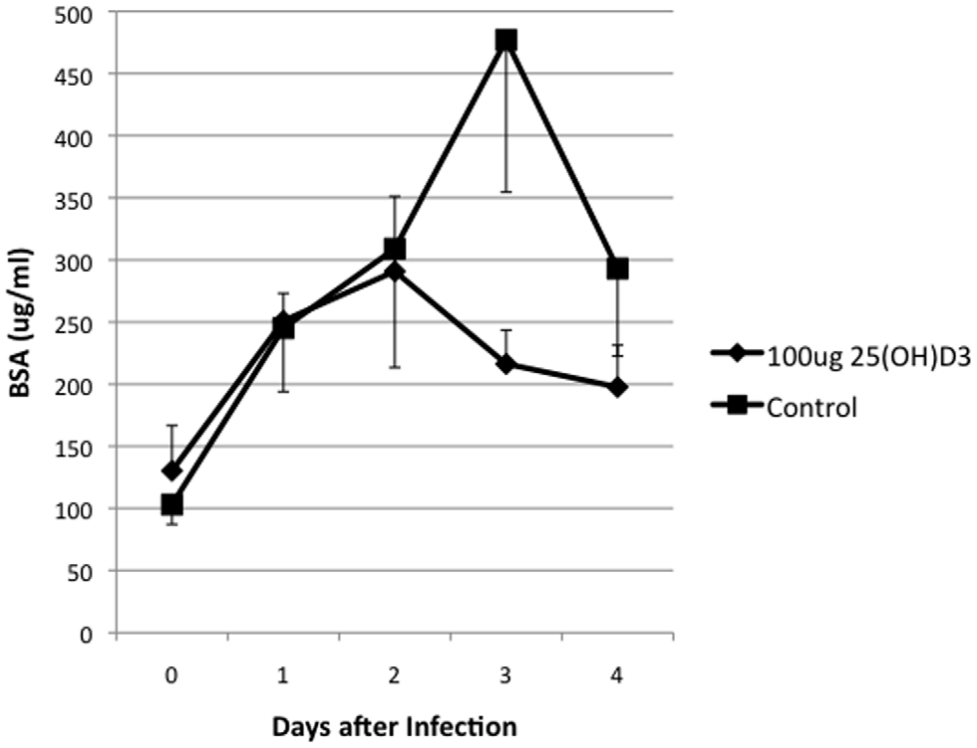
Bovine Serum Albumin in Milk of Control and 25(OH)D_3_ Treated Animals. Milk samples were tested for BSA levels at each time point and the average was determined for 25(OH)D_3_ treated (υ) and control animals(ν).

Additional indicators of a mammary gland infection were monitored, including rectal temperatures, somatic cell counts, and BSA in the milk. Rectal temperatures showed a 25(OH)D_3_ treatment effect with a *P*  = 0.065 and are plotted in figure 2. There was a continuation of the trend that the 25(OH)D_3_ treated group had a better clinical outcome, in that somatic cell counts were lower in the 25(OH)D_3_ treated group (figure 3). Acute bacterial infections are known to increase mammary vascular permeability, an indicator of this change is increased BSA levels in milk [27]. Milk from the morning milking was tested by ELISA for BSA (figure 4). Day 3 levels of BSA were higher in the control animals compared to the 25(OH)D_3_ treated animals (*P*  = 0.07).

**Figure 5:**
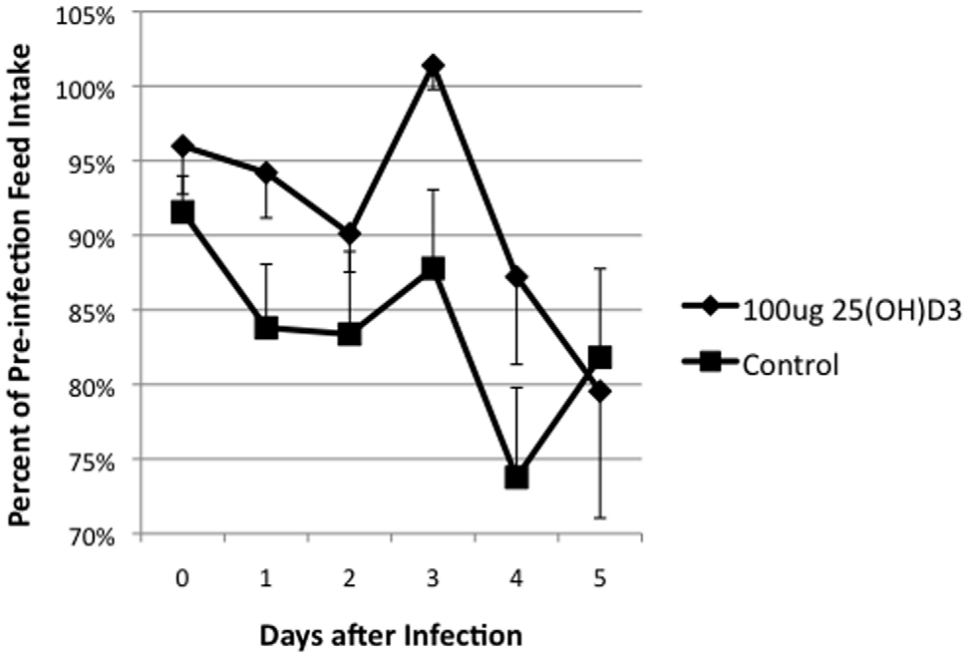
Feed Intake in Control and 25(OH)D_3_ Treated Animals. Daily feed intake was determined for each cow. Data are expressed as a percentage of the pre-infections (the average of the 4 days feed intake prior to infection). Each the average was determined for 25(OH)D_3_ treated (υ) and control animals(ν).

**Figure 6:**
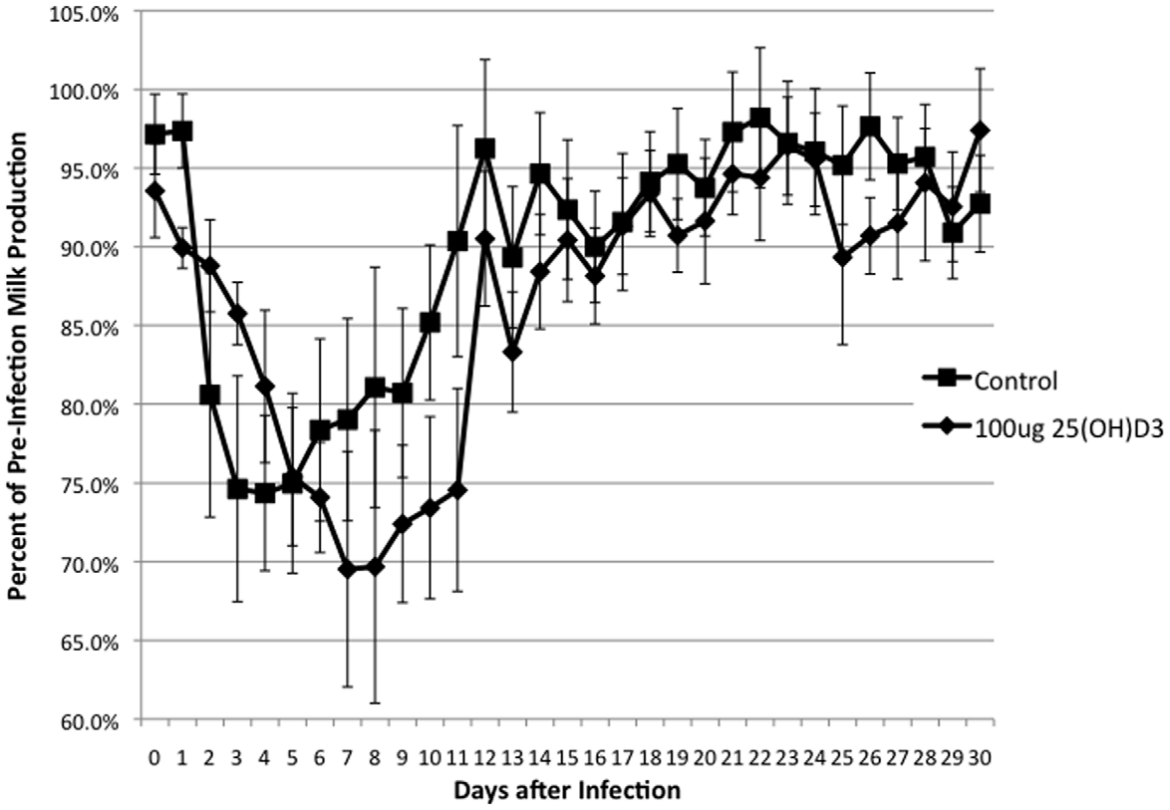
Milk Production in Control and 25(OH)D_3_ Treated Animals. Daily milk production was determined for each cow. Data are expressed as a percentage of the pre-infections (the average of the 4 days milk production prior to infection). Each the average was determined for 25(OH)D_3_ treated (υ) and control animals(ν). Repeated measures analysis showed a significant treatment x time effect.

Mastitis causes reduction of both feed intake and milk productions, and the level of reduction correlates with the severity of the infection. The pre-infected feed intake and milk production was calculated as the average of the values from the four days prior to the infection. There was a trend for the 25(OH)D_3_ treated animals to have higher average feed intake (figure 5). There was a significant (*P* <0.05) time x treatment effect, as the 25(OH)D_3_ treated animals milk production decline due to the infection occurred later in the infection compared to control cows (figure 6).

Table 1 shows the blood serum levels of 25(OH)D_3_ in the control animals compared to the 25(OH)D_3_ treated animals. This data demonstrates that the dose of 100 ug 25(OH)D_3_ twice daily did not affect (*P*>0.10) blood serum 25(OH)D_3_ levels.

**Table 1:** Serum levels of 25(OH)D_3_ in the cows treated with 100 ug 25(OH)D_3_ infused into the mammary gland and cows untreated.

	No treatment (ng/ml)	25(OH)D3 (ng/ml)
Day 0	50.9±3.0	47.7±4.8
Day 4	52.6±7.5	54.3±7.5
Day 10	60.0±8.3	59.7±9.8

Numbers represent the mean±standard error of the mean.

## Discussion

In a recent review it was stated that “Data from several models of infection have so far not supported a role of vitamin D in affecting the course of disease” [28]. These authors' conclusions are based on 1) the lack of in vivo evidence for an effect of vitamin D status on the course of disease and 2) the findings that 1, 25(OH)_2_D_3_ inhibits T helper cell functions that are important in many infections and which we have characterized in vitro using a cow model [29]. The data presented in this study demonstrate that in vivo administration of 25(OH)D_3_, used as a treatment, reduces the severity of an intramammary infection. The effectiveness of 25(OH)D_3_ may be due to many factors, including a predominant role of the innate immune response in mastitis and that the milk normally has low 25(OH)D_3_ levels. Monocytes/macrophages play a critical role in the immune response to mastitis [30] and intracrine produced 1,25(OH)2D3 effects many aspects of the innate immune system [31] and specifically macrophages antimicrobial mechanisms [2], [5]. Our ability to demonstrate in vivo efficacy of 25(OH)D_3_ on a bacterial infection (figure 1) may be due to the fact that the milk compartment of the mammary gland is relatively devoid of 25(OH)D_3,_ even though systemic vitamin D status is excellent (table 1). Concentrations of 25(OH)D_3_ in milk are only 0.3–0.6 ng/ml compared to 35 ng/ml in serum considered necessary for full immune function [17], [18], [25]. It may be that treatment with 25(OH)D_3_ in individuals with sufficient circulating 25(OH)D_3_, will only be effective for infections in anatomical locations with low concentrations of 25(OH)D_3_, such as the mammary gland, and possilby the lung, and upper respiratory tract. It is thus important to note that the data presented here did not directly address the broader issue of “systemic vitamin D status” on the course of disease. However, these data clearly demonstrate that increasing 25(OH)D_3_ concentrations in a tissue with low 25(OH)D_3_ concentrations can positively influence the early course of the disease.

In this study, the treatment of an intramammary infection with 25(OH)D_3_ reduced bacterial counts (figure 1), decreased the severity of the disease (figures 2, 3, 4, 5), and delayed loss of milk production (figure 6). Based on human studies [16] these results would depend on the ability of activated monocytes/macrophages to produce 1,25(OH)_2_D_3_ from 25(OH)D_3_ and to induce antibacterial peptides such as cathelicidin. Bovine monocytes/macrophages produces 1,25(OH)_2_D_3_ from 25(OH)D_3_ both in vitro [4] and in vivo [3] following bacterial activation. Unlike human monocytes, bovine moncytes stimulated with 1,25(OH)_2_D_3_ does not lead to the induction of antibacterial cathelicidins in the cattle [4]. To date, only a few genes in bovine have been identified as responsive to 1,25(OH)_2_D_3_ , namely, nitric oxide synthetase , the chemokine RANTES, vitamin D receptor, S100 calcium binding protein A12 (S100A12), and 24-hydroxylase [3], [4], [29]. Presumably other immune mediators are involved in the vitamin D immune pathway and experiments to determine them are ongoing. We do not know whether the lower bacterial counts in the 25(OH)D_3_ treated animal are the result of enhanced nitric oxide killing, specific leukocyte recruitment, and/or production of a yet unidentified antibacterial peptide.

Administration of 25(OH)D_3_ is known to affect the innate immune system [2], [17], [31]. In the case of humans 1,25(OH)_2_D_3_ treatment can cause increased expression of cathelicidins or defensins [32], and in the case of cattle 1,25(OH)_2_D_3_ treatment can cause increased expression in nitric oxide and RANTES [4]. Since the first significant affect of 25(OH)D_3_ treatment was seen at 48 hours (fourth milking) of infection, it is likely that the affect of 1,25(OH)_2_D_3_ treatment is on the innate immune system. However, we have recently shown that the bovine adaptive immune system is also sensitive to 25(OH)D_3_ treatment [29]. In those experiments, cattle were immunized with an antigen several weeks prior to the experiment. We showed that antigen-stimulated PBMC from those immunized cattle, when treated with antigen and 25(OH)D_3_ suppressed IFN-γ and IL-17F in T cells. The role of T cells in bovine mastitis is not well defined, however, reinfection of animals treated and not treated with 25(OH)D_3_ would begin to assess the role of the affect of vitamin D on the adaptive immune system during an infection.

At the end of this experiment all animals were treated with antibiotics to eliminate the *S. uberis* infections indicating that 25(OH)D_3_ treatment alone was not an effective in eliminating the infection. However, the reduction in the number of bacteria and severity of disease shown in this study suggests that 25(OH)D_3_ may be effective in combination with antibiotics. This combined-treatment approach may allow for reductions in antibiotic use and diminish concerns about antibiotic residues in the dairy products and development of antibiotic resistance in food animals. Although not evaluated in the present study a combined antibiotic and vitamin D therapy may provide an effective treatment strategy for chronic infections that are not effectively treated by antibiotics alone.

In conclusion we demonstrated for the first time a positive in vivo effect of intramammary administration of 25(OH)D_3_ on the course of a bacterial infection in the mammary gland. This finding suggested a significant non-antibiotic complimentary role for 25(OH)D_3_ in the treatment of infections in compartments naturally low in 25(OH)D_3_ such as the mammary gland and by extension, a potentially useful treatment of lung/respiratory tract infections via aerosol administration. These experiments were designed to focus on the early innate immune response to experimental intramammary infection and do not address to affect of 25(OH)D_3_ on adaptive immunity to bacterial infections. Further studies will be needed to address these important questions.
